# A Comprehensive Physiotherapeutic Approach in a Young Patient With Necrotizing Pancreatitis With Bilateral Pleural Effusion: A Case Report

**DOI:** 10.7759/cureus.54524

**Published:** 2024-02-20

**Authors:** Anushri R Patil, Lajwanti Lalwani

**Affiliations:** 1 Department of Cardiorespiratory Physiotherapy, Ravi Nair Physiotherapy College, Datta Meghe Institute of Higher Education and Research, Wardha, IND

**Keywords:** six-minute walk test, quality of life, physiotherapy, pleural effusion, necrotizing pancreatitis

## Abstract

Necrotizing pancreatitis represents a severe variant of acute pancreatitis characterized by the death of pancreatic tissue (necrosis). This condition commonly stems from inflammation and damage to the pancreas, leading to the development of areas of dead tissue within the organ. Pleural effusion, on the other hand, involves the accumulation of fluid within the pleural cavity. Typically, these effusions are of mild to moderate severity and tend to occur on the left side. In the following case report, we present a 25-year-old male who was diagnosed with necrotizing pancreatitis and bilateral pleural effusion. It is important to emphasize that cardiopulmonary physiotherapy plays a crucial role in managing pleural effusion. Such interventions, which encompass breathing exercises and thoracic expansion exercises, are pivotal for optimizing lung ventilation, enhancing oxygen levels, and preventing complications such as atelectasis and pneumonia. By boosting oxygenation and improving lung compliance, physiotherapy helps reduce the risk of respiratory problems and expedites the recovery process. This approach enables young individuals to regain their lung function and overall quality of life. In this particular case, the patient received medical management and pulmonary rehabilitation, resulting in a decrease in the Modified Medical Research Council Scale score and an improvement in the six-minute walk test (6 MWT), which subsequently enhanced their quality of life.

## Introduction

Acute pancreatitis is characterized by an abrupt onset of pancreatic inflammation, which has the potential to result in pancreatic tissue destruction, a condition known as pancreatic necrosis. Necrotizing pancreatitis is a delayed complication of acute pancreatitis. It is distinguished by the death of the pancreatic parenchyma, which may be accompanied by necrosis of the surrounding peripancreatic tissues. Pancreatic necrosis is a potentially fatal condition that can lead to organ failure in other parts of the body, such as the lungs and kidneys [1]. Several key symptoms distinguish necrotizing pancreatitis. First and foremost, people frequently experience abdominal pain, which is usually located in the epigastric or left upper quadrant of the abdomen. While this type of pain is not unique to this condition, it may spread to the back, flank, or chest. The intensity of the pain varies, but it is commonly described as dull and cramp-like. Other common symptoms include fever, vomiting, abdominal swelling, and dehydration. Magnetic resonance imaging (MRI) and computed tomography (CT) scans are commonly used by doctors to diagnose necrotizing pancreatitis [2]. The factors behind necrotizing pancreatitis align closely with those of acute pancreatitis, with gallstone disease ranking as the most frequent cause. Following closely the second most common cause of necrotizing pancreatitis is alcohol consumption [3].

Pleural effusion is a common late complication of necrotizing pancreatitis. It can be seen both during admission and during the hospital stay [4]. In acute necrotizing pancreatitis cases, respiratory issues are observable either through clinical examination or radiographic assessments in approximately 33% of patients. Among the pulmonary complications associated with acute necrotizing pancreatitis in this group are pulmonary infiltrates or atelectasis (15%), pleural effusions (ranging from 4% to 17%), and pulmonary edema (ranging from 8% to 50%) [5,6]. Pleural effusion is now considered a sign of severe pancreatitis rather than just a marker of the disease [6,7]. In acute pancreatitis, pleural effusions are usually characterized by their small size and blood-tinged appearance. They exhibit elevated amylase levels (often reaching up to 30 times higher than the corresponding serum value), increased protein content (exceeding 30 grams/Liter), and a higher ratio of lactic acid dehydrogenase (LDH) compared to the serum value (greater than 0.6).

A large portion of pleural effusions (68%) occur on the left side, while 22% are bilateral, and the remaining 10% are exclusively on the right side. The leading causes of pleural effusion predominantly involve either a blockage of lymphatic drainage through the diaphragm or the development of pancreaticopleural fistulas due to leaks or disruptions in the pancreatic duct, often stemming from an episode of acute pancreatitis [6,8]. Initially, a conservative approach is commonly employed in the treatment of pleural effusions. However, when pleural effusions cause symptoms, they often require interventions such as thoracentesis, tube thoracotomy, endotracheal intubation, admission to the intensive care unit (ICU), parenteral nutrition, and the administration of octreotide. It is noteworthy that pleural effusions often subside as the underlying intraabdominal cause is resolved [6,8]. Physiotherapy has been suggested as an adjunct therapy alongside both surgical and non-surgical treatments. This is a vital intervention aimed at preventing and alleviating the adverse consequences of extended bed rest during hospitalization, while simultaneously enhancing respiratory function. Pulmonary rehabilitation primarily encompasses a range of activities such as breathing control exercises, lung expansion exercises, relaxation exercises, postural exercises, mobilizations, and educational components as part of respiratory physiotherapy. Pulmonary rehabilitation is an integral aspect of managing respiratory conditions and is recommended to be used in conjunction with medical treatment for improved outcomes [9,10].

## Case presentation

A 25-year-old male patient, an employee by occupation, came with complaints of abdominal pain, abdominal fullness for 20 days, difficulty in breathing, and fever for five days. He visited a local hospital where investigations like blood routine and ultrasonography (USG) were done and the patient was diagnosed with necrotizing pancreatitis. The patient took medication for the same. When the patient stopped medication, abdominal pain aggravated along with breathlessness which was gradual in onset, which was grade II on Modified Medical Research Council (MMRC), for which the patient reported to the hospital. The patient underwent investigations like X-ray and USG and he was diagnosed with necrotizing pancreatitis with bilateral pleural effusion. Later, he was managed with medications and further referred for cardiorespiratory physiotherapy.

Clinical findings

The examination was done after obtaining informed consent from the patient and relative. The patient was assessed in a supine lying position with the head end elevated in a well-lit room. The patient was conscious and well-oriented to time, place, and person and obeyed commands. The patient was mesomorphic in build with a body mass index (BMI) of 23.9 Kg/m^2^ which is considered normal. On physical examination, the patient complained of breathlessness of grade II on the MMRC scale. Foley’s catheter was in situ. The patient was on 4 liters of 02 via a face mask. The patient’s pulse rate was 76 beats per minute, the respiratory rate was 21 breathes per minute, and blood pressure was 140/70 mmHg without orthostatic changes. On palpation, tenderness was present over the abdominal region, grade 1 i.e. the patient complained of pain. On auscultation, air entry was reduced at bilateral lower zones. On percussion, there was a dull note over bilateral lower zones. Tactile vocal fremitus and vocal resonance revealed decreased resonance over bilateral lower zones. Cardiovascular and respiratory examination revealed that apex impulse was present at the fifth intercostal space, and chest expansion was reduced at the nipple level and xiphisternum level.

Diagnostic investigation

**Figure 1:**
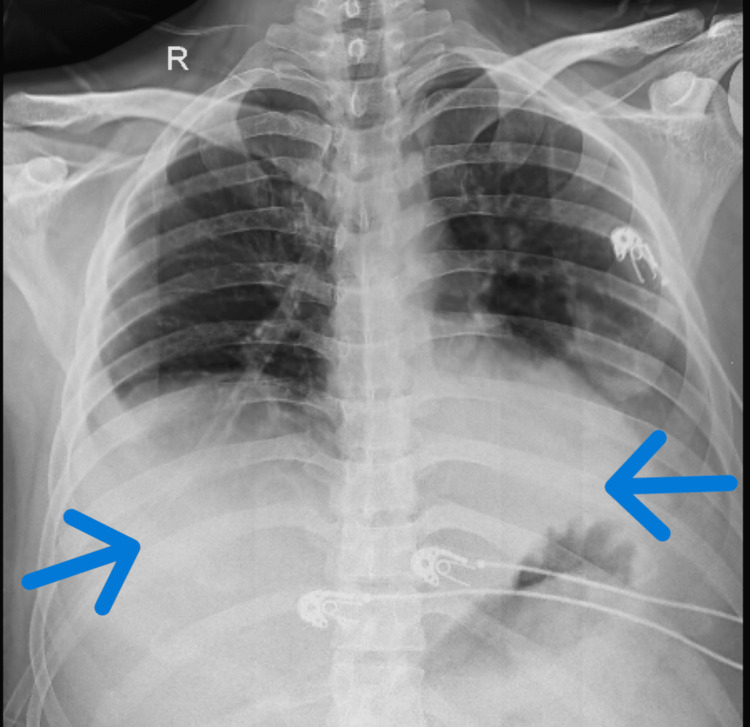
Radiograph (postero-anterior view) revealing pleural effusion on left > right side Homogeneous density is seen in lower zones, diminished cardio-phrenic and costophrenic angle. The accumulation of fluid highlighted with arrows at right and left lower zones.

USG revealed bilateral pleural effusion left > right while the right lung had approximately 800-1000 cubic centimeters and the left lung had 1000-1200 cubic centimeters fluid. Arterial blood gas (ABG) analysis (pre-physiotherapy intervention) was fully compensated by metabolic alkalosis, and ABG analysis (post-physiotherapy intervention) was normal. Figure 1 shows the radiograph of the lung revealing pleural effusion.

Physiotherapy management 

An advanced physiotherapy protocol was planned for the patient for five weeks, followed by a home program of two weeks (Tables 1, 2). Segmental breathing exercises, which target specific lung areas, resulted in significantly increased chest expansion, particularly in the middle and lower lungs. These exercises stretch and improve the function of the muscles between the ribs, making them more effective at managing pleural effusion. Active expiration lengthens the diaphragm, allowing it to operate close to its optimal length. Incentive spirometry was utilized as a lung expansion therapy. Limb exercises (passive, active assisted, or active) were performed in a seated position based on medical advice to maintain joint range of motion, improve soft tissue length, muscle strength, and function, reduce the risk of thromboembolism, and improve endurance [10].

**Table 1:** Tailored physiotherapy management which was given for three weeks

Goal	Intervention	Description of intervention	Repetitions
Patient education	Guidance to patient and patient’s relative	Cause of pleural effusion are taught Importance of pulmonary rehabilitation along with medical management is given Patient is sensitized about significance of pulmonary rehabilitation, adherence to exercise program	-
To improve ventilation and oxygenation of lung	Segmental breathing exercise such lateral coastal expansion, incentive spirometry Deep breathing.	The patient is instructed to inhale and exhale while exhaling physiotherapist applies pressure laterally and downward.	10 repetitions X 1 set
To improve thoracic expansion	Thoracic expansion exercises	The patient is instructed to lift hands while inhaling in and bring hands down while exhaling as it helps to increase chest expansion.	10 repetitions X 1 set
To induce relaxation	general relaxation techniques psychological support	Relaxation with the help of the Jacobsons technique	15 mins
To improve flexibility	Upper limb and lower limb mobility exercise	The patient is instructed for active range of motion exercise	10 repetitions X 1 set
To improve endurance	Spot marching exercise, ambulation around hall	The patient is instructed to do spot march beside bed, and ambulation around hall is advised further progressed to stairs in presence of physiotherapist.	15 minutes
To increase strength of muscle	Upper limb and lower limb strengthening exercises using ½ kg weight cuff.	The patient is instructed to do active range of motion exercise with help of weight cuff	10 repetitions X 1 set

**Table 2:** Advanced pulmonary rehabilitation which was given for two weeks

Goal	Intervention	Description of intervention	Repetitions
Aerobic training	Arm/leg ergometry	The patient is instructed about static cycle and its working. He instructed to do cycling until he feels fatigued	30 minutes
To improve inspiratory muscle strength and pulmonary function.	Inspiratory muscle training device	The patient is instructed to inhale as deep as possible in the device and exhale through it. It is performed under physiotherapist supervision.	20 repetitions X 2 sets
Strength training	Resistance training with the help of theraband	The patient is advised to perform exercises with help of theraband providing resistance to improve strength of upper limb and lower limb muscles.	20 repetitions X 2 sets
Home program	Breathing exercises like deep breathing exercises. Strengthening for upper limb and lower limb exercise with the help of a 1-liter water bottle. Incentive spirometry Walking for endurance	All exercises are prescribed for home program so as to prevent collapse.	20 repetitions X 2 sets

Follow-up and outcome measure

The patient was thoroughly assessed before patient rehabilitation, outcome measures were recorded post-rehabilitation, the home program was prescribed for two weeks, and assessment was taken at the time of follow-up (end of the seventh week) which revealed progression. Table 3 shows the outcome measures that were assessed after the completion of advanced pulmonary rehabilitation. The reliability of the six-minute walk test is 0.96 and the validity is 0.88.

**Table 3:** Outcome measures SF-36: Short form health survey; MMRC: Modified Medical Research Council

Outcome measure	Pre-physiotherapy intervention	Post-physiotherapy intervention (5^th^ week)	Follow-up (7^th^ week)
MMRC scale of dyspnea	Grade II	Grade I	Grade I
Six-minute walk test	120 m	370 m	450 m
Health-related quality of life (SF-6)	20	46	55

**Figure 2:**
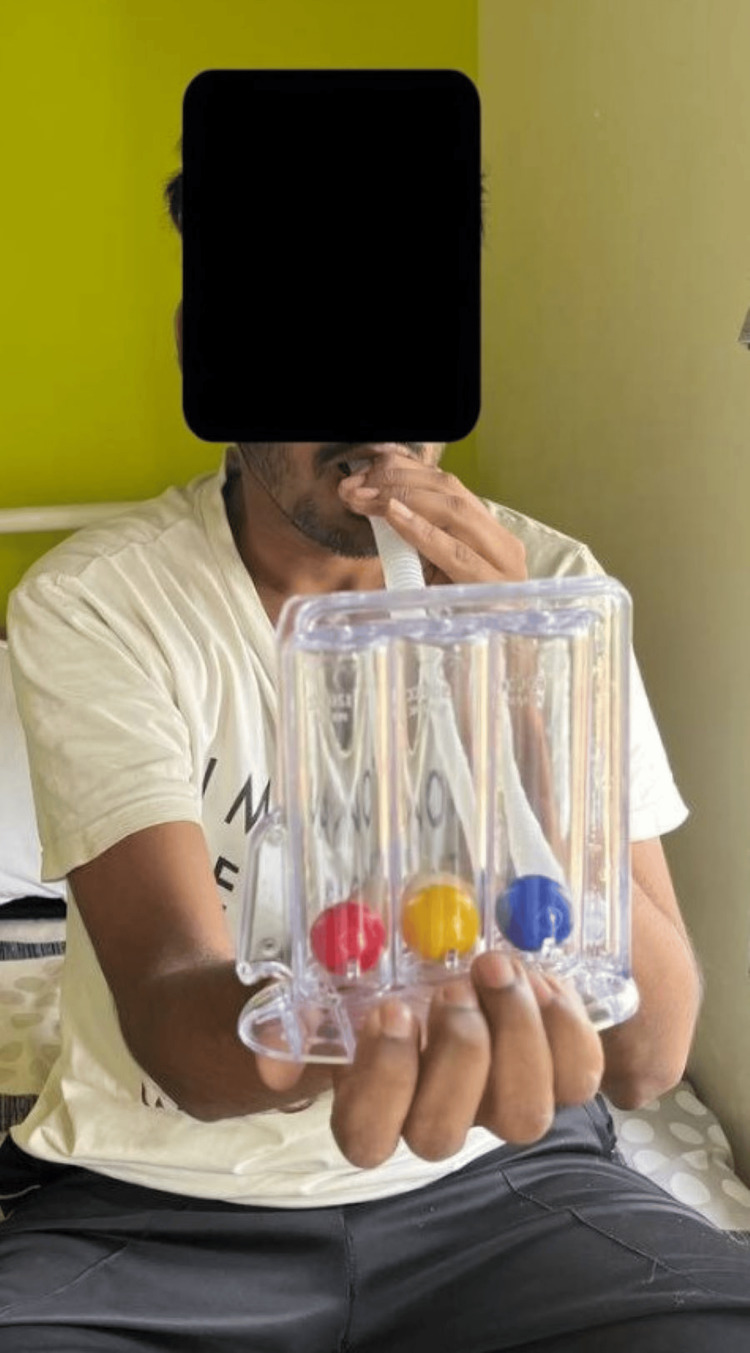
Patient performing incentive spirometry

Figure 1 and Figure 2 show the patient performing physiotherapy exercises. 

**Figure 3:**
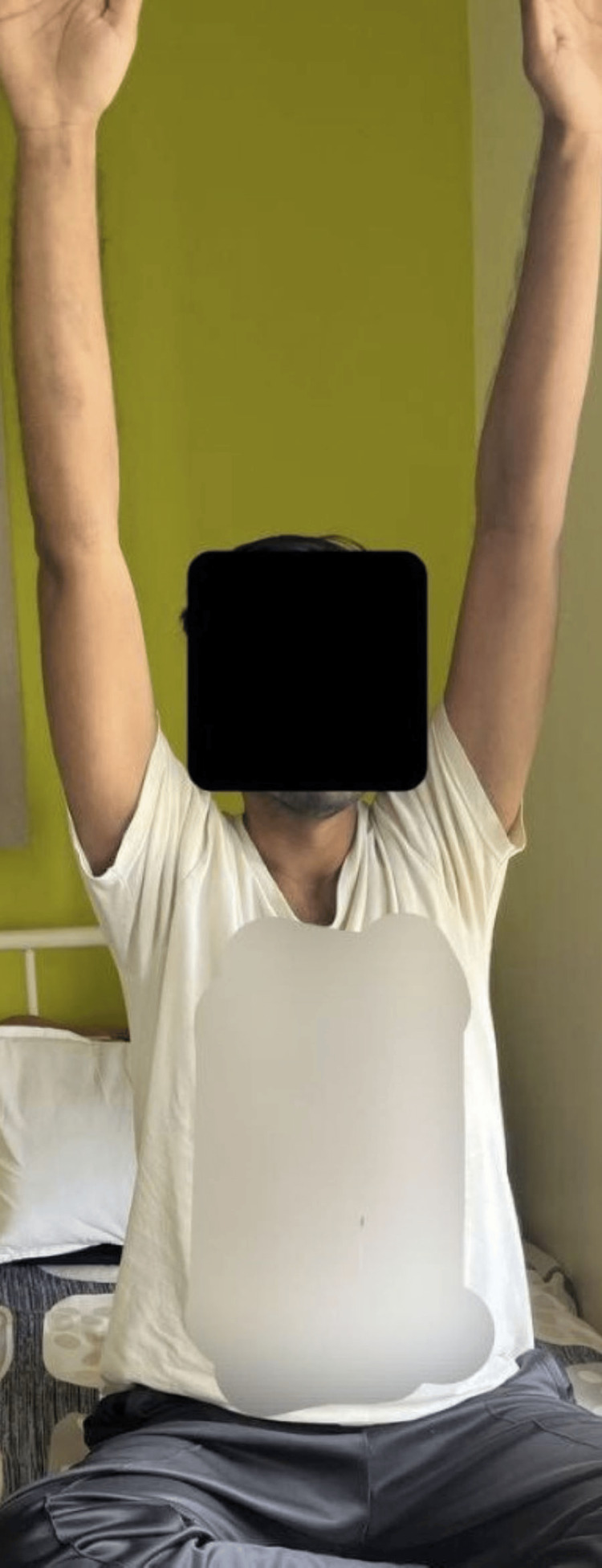
Patient performing thoracic expansion

## Discussion

Pleural effusion is the accumulation of fluid in pleural spaces in the lungs. Symptoms like cough and breathlessness can be one of the early signs of pleural effusion. It can be with of help of medications or with the help of the thoracocentesis procedure. physiotherapy plays an important role in adjuvant medical treatment. It is important to improve lung capacity, endurance, and aerobic capacity, which is affected by pleural effusion. Mobilization techniques and exercises, deep breathing exercises, and incentive spirometry were all part of the physiotherapy protocol. We incorporated breathing exercises such as segmental breathing exercises and lateral coastal expansion exercises which helped to improve lung expansion. Incentive spirometry is used to prevent lung collapse and improve breathing. Endurance exercises were incorporated to prevent the ill effects of hospital stay. We have incorporated advanced pulmonary rehabilitation to improve the respiratory efficiency and overall endurance of the patient. we found greater improvement in patient outcome measures. A study conducted by Valenza-Demet et al. showed improvement in patients with pleural effusion after using exercises for strength, flexibility, range of motion, and endurance [11,12]. In a study conducted by dos Santos et al., they studied the effect of physiotherapy techniques in pleural disease to hassle pleural drainage. They used techniques like incentive spirometry, airway clearance exercises, and devices for pulmonary rehabilitation in pleural effusion [13,14]. Gunjal et al. conducted a study in which they studied the effect of deep breathing versus segmental breathing in patients with pleural effusion and found segmental breathing has a significant effect in improving lung functions [15].

Exercises focusing on chest mobility enhance the flexibility of the chest wall, trunk, and shoulder region, as well as the depth of breath. Such exercises are valuable for pleural effusion patients aiming to enhance their chest expansion [16]. In their research, Tahir and colleagues found that segmented breathing exercises are more advantageous and effective in enhancing chest expansion and lung function in pleural effusion patients compared to deep breathing exercises [9,17]. In a study conducted by Sanchez-Ramirez, they studied the effect of various pulmonary rehabilitation services on a patient with a restrictive and obstructive pulmonary condition like a six-minute walk test and health-related quality of life short-form health survey (SF-36) using pulmonary rehabilitation and they found increased in six-minute walk test levels and health-related quality of life (SF-36) scores [18]. According to a study conducted by Tiwari et al., they found pulmonary rehabilitation adjunct to medical treatment of alcoholic patients with pleural effusion proved to decrease dyspnea with the help of an MMRC scale of dyspnea [19]. Furthermore, as part of the larger medical team, this case report emphasizes the importance of early intervention, advanced pulmonary rehabilitation, and ongoing monitoring by a skilled physiotherapist. Timely and appropriate physiotherapy interventions not only relieve respiratory distress but also contribute to a shorter hospital stay and lower healthcare costs [20].

## Conclusions

Subsequently, this case report demonstrates the critical role of physiotherapeutic rehabilitation in the treatment of a young patient with necrotizing pancreatitis complicated by bilateral pleural effusion. The patient did not have any adverse effects from the treatment. The successful outcome in the MMRC dyspnea scale, increase in six-minute walk test scores, and improvement in health-related quality of life (SF-36) scores highlight the importance of a multidisciplinary approach to the care of such complex medical conditions. Thus, tailoring physiotherapy interventions to each patient's specific needs and condition remains critical. Physiotherapeutic rehabilitation is an important addition to the treatment of patients with necrotizing pancreatitis complicated by pleural effusion, providing a comprehensive approach to recovery and improved overall well-being. As this was a case report, the study was done on a single patient; in the future, more studies need to be done on more patients.
